# Habitat Loss in the IUCN Extent: Climate Change-Induced Threat on the Red Goral (*Naemorhedus baileyi*) in the Temperate Mountains of South Asia

**DOI:** 10.3390/biology13090667

**Published:** 2024-08-27

**Authors:** Imon Abedin, Tanoy Mukherjee, Joynal Abedin, Hyun-Woo Kim, Shantanu Kundu

**Affiliations:** 1 Agricultural and Ecological Research Unit, Indian Statistical Institute, Kolkata 700108, India; imon.jabedin@gmail.com (I.A.); mukherjeetanoy@gmail.com (T.M.); 2 Dibru-Saikhowa Conservation Society, Tinsukia 786147, India; banashree.eco@gmail.com; 3 Department of Marine Biology, Pukyong National University, Busan 48513, Republic of Korea; 4 Marine Integrated Biomedical Technology Center, National Key Research Institutes in Universities, Pukyong National University, Busan 48513, Republic of Korea; 5 Department of Biology, Faculty of Science and Technology, Airlangga University, Surabaya 60115, Indonesia; 6 Institute of Fisheries Science, College of Fisheries Science, Pukyong National University, Busan 48513, Republic of Korea; 7 International Graduate Program of Fisheries Science, Pukyong National University, Busan 48513, Republic of Korea

**Keywords:** artiodactyla, ecological niche, global warming, international conservation, threatened species, transboundary PAs

## Abstract

**Simple Summary:**

Global warming has had a dramatic impact on many mammalian species, leading to declines and even extinctions, particularly in high-altitude regions of the Himalayas. The Red Goral, a cliff-dwelling ungulate with a restricted range in India, Myanmar, and China, is especially vulnerable due to its small population and limited habitat. Recent habitat modeling indicates that only a small portion of its extent is currently suitable, with even less protected within conservation areas. The projections for the future are concerning and reveal significant declines in suitable habitats and increased fragmentation. The key factors affecting the species distribution were found to be precipitation and elevation. The study highlights the importance of maintaining and improving connectivity between fragmented habitats and identifies crucial protected areas for conservation efforts. The recommendations stress the need for strategic management, enhanced international cooperation, and addressing traditional hunting practices to better protect the species. Overall, the findings underscore the urgent need for effective conservation strategies to ensure the long-term survival of the Red Goral in its natural environment.

**Abstract:**

Climate change has severely impacted many species, causing rapid declines or extinctions within their essential ecological niches. This deterioration is expected to worsen, particularly in remote high-altitude regions like the Himalayas, which are home to diverse flora and fauna, including many mountainous ungulates. Unfortunately, many of these species lack adaptive strategies to cope with novel climatic conditions. The Red Goral (*Naemorhedus baileyi*) is a cliff-dwelling species classified as “Vulnerable” by the IUCN due to its small population and restricted range extent. This species has the most restricted range of all goral species, residing in the temperate mountains of northeastern India, northern Myanmar, and China. Given its restricted range and small population, this species is highly threatened by climate change and habitat disruptions, making habitat mapping and modeling crucial for effective conservation. This study employs an ensemble approach (BRT, GLM, MARS, and MaxEnt) in species distribution modeling to assess the distribution, habitat suitability, and connectivity of this species, addressing critical gaps in its understanding. The findings reveal deeply concerning trends, as the model identified only 21,363 km^2^ (13.01%) of the total IUCN extent as suitable habitat under current conditions. This limited extent is alarming, as it leaves the species with very little refuge to thrive. Furthermore, this situation is compounded by the fact that only around 22.29% of this identified suitable habitat falls within protected areas (PAs), further constraining the species’ ability to survive in a protected landscape. The future projections paint even degraded scenarios, with a predicted decline of over 34% and excessive fragmentation in suitable habitat extent. In addition, the present study identifies precipitation seasonality and elevation as the primary contributing predictors to the distribution of this species. Furthermore, the study identifies nine designated transboundary PAs within the IUCN extent of the Red Goral and the connectivity among them to highlight the crucial role in supporting the species’ survival over time. Moreover, the Dibang Wildlife Sanctuary (DWLS) and Hkakaborazi National Park are revealed as the PAs with the largest extent of suitable habitat in the present scenario. Furthermore, the highest mean connectivity was found between DWLS and Mehao Wildlife Sanctuary (0.0583), while the lowest connectivity was observed between Kamlang Wildlife Sanctuary and Namdapha National Park (0.0172). The study also suggests strategic management planning that is a vital foundation for future research and conservation initiatives, aiming to ensure the long-term survival of the species in its natural habitat.

## 1. Introduction

In recent years, global warming has significantly impacted numerous vertebrate species, including mammals, leading to their rapid decline or even extinction within their ecological niches [1,2]. These ecological niches are crucial for species survival and their interactions with environmental conditions, promoting viable populations and their persistence over time [3]. The rapid decline of these ecological niches is expected to worsen as the pace and magnitude of global environmental warming continue to accelerate [4]. This is more concerning for mountainous and temperate regions because these areas are highly sensitive to temperature changes, which can lead to rapid and significant ecological disruptions as even small increases in temperature can cause accelerated snowmelt, shifts in vegetation zones, and habitat loss, creating more immediate and severe impacts compared to more stable lowland environments [5,6]. Mountain mammals cope with thermic stress and decreasing suitable areas by shifting to higher altitudes, altering their activity patterns, seeking cooler microhabitats, adjusting their diet, and modifying reproductive strategies, while conservation efforts focus on expanding protected areas and improving habitat management to support their adaptation [7,8]. Therefore, this situation is particularly pronounced in remote and highly elevated mountainous regions, such as the Himalayas and adjacent ranges in South Asia [1]. The continental drift theory suggests that the Indian and Eurasian plates collided around 50 million years ago, forming the Himalayas, also known as “the abode of snow” in Sanskrit, and creating a complex geological wedge with three tectonic domains from north to south [9,10]. The Himalayan range is divided into three sections: the western, central, and eastern ranges, each home to unique flora and fauna, including many mountainous ungulates. Unfortunately, many of these species lack adaptive strategies to cope with new climatic conditions [11,12,13]. The eastern Himalayan range, in particular, suffers from limited research and adaptive conservation strategies due to its remoteness, challenging terrain, and inadequate infrastructure. This leaves many species understudied, necessitating the identification of suitable areas where large ungulates with narrow niches can find refuge under future climatic conditions to improve conservation efforts [14,15]. This strategic approach is of utmost importance for the Red Goral (*Naemorhedus baileyi*), a cliff-dwelling Bovidae species (order Artiodactyla) classified as “Vulnerable” by the IUCN Red List due to its small and declining population [16]. Morphologically, this species is a vibrant foxy-red mammal characterized by its long, soft, shaggy hair, with a thin, dark stripe running along its back from head to tail [17]. The legs of this species exhibit the same rich red hue as the rest of its body, while the undersides are a lighter buff color. The Red Goral has the most restricted range of all goral species, confined to the temperate mountains of northeast India (Arunachal Pradesh), northern Myanmar, and China (southeast Tibet and Yunnan). This is the only goral species in this range and inhabits higher elevations than most gorals, found between altitudes of 2000 and 4500 m [16].

Since this species has an extremely restricted range and a very small population size, its vulnerability to climatic shifts and habitat alterations is markedly increased, making it particularly susceptible to changes in environmental conditions and disruptions to its habitat [18]. Therefore, habitat mapping and modeling corridors across the species’ distribution are essential for prioritizing conservation strategies [19]. These efforts will demonstrate the suitability and viability of habitat patches for wildlife occupancy and movement between them, thereby facilitating natural gene flow and enhancing long-term survival [20,21]. Furthermore, identifying the key factors driving shifts in species’ distribution ranges due to climatic changes is crucial, as understanding these drivers will offer insight into the environmental alterations that impact habitat preferences and movement patterns, ultimately guiding the development of effective conservation and management strategies [22,23].

In recent years, there have been significant advances and widespread adoption of species distribution models (SDMs) for assessing habitat suitability [24,25]. This methodology facilitates the mapping of species distribution patterns and the quantitative evaluation of various environmental factors [25,26,27,28]. Consequently, the ensemble model has emerged as a robust tool for estimating habitat suitability across different species [29]. This approach integrates multiple modeling algorithms to predict species distributions across geographic areas, leveraging the strengths of various models to capture diverse aspects and underlying processes influencing distribution [30]. The integrated approach combines various modeling methods, each capturing different aspects and underlying processes that influence species distribution. This strategy seeks to balance the strengths and limitations of individual models, leading to improved prediction accuracy and reliability. Furthermore, understanding the response of key driving variables to climate change is essential for identifying suitable habitats, which is critical for developing effective conservation strategies and landscape-level management plans [31,32]. Moreover, studying the IUCN extent is justified as it involves a systematic process that includes mapping and analyzing the geographical distribution of a species using occurrence records, adjusting for geographical barriers, and verifying with expert knowledge to accurately represent the species’ natural distribution for conservation planning and risk assessment.

Therefore, this study utilizes an ensemble approach in SDM within the IUCN range of the Red Goral, *N. baileyi*, to (i) determine the suitable extent under current and future climate change scenarios; (ii) analyze the patterns of habitat fragmentation resulting from climate change; (iii) identify biological corridors within the range; and (iv) assess the suitability and connectivity of transboundary protected areas (PAs). This study is vital for enhancing conservation efforts for this species that lacks extensive research and faces vulnerability in the ecological context. The identification of suitable habitats under current and future climate scenarios will reveal the areas in which this species can thrive within the IUCN extent and the habitat shifts with changing conditions to guide conservationists in India and adjoining countries. Moreover, understanding patterns of habitat fragmentation and connectivity will aid in safeguarding these corridors that will enable movement for this species and ensure transboundary migration and genetic connectivity within the known range. Furthermore, evaluating the suitability and connectivity of PAs will ensure coordinated conservation efforts across borders, creating an international network of safe havens and supporting broader conservation initiatives to save this threatened ungulate species in the wild.

## 2. Materials and Methods

### 2.1. Study Area and Species Occurrence Data

**Figure 1:**
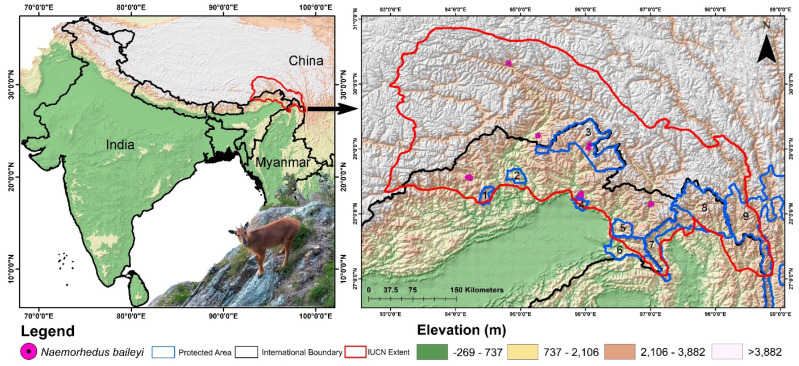
Map showing the study area for the present study along with the IUCN extent of Red Goral (*N. baileyi*). The figure also highlights the location points acquired from primary and secondary sources used for training the model. The photograph of the Red Goral was taken by Mr. Ravi Mekola in Dibang Valley, Arunachal Pradesh, India. Protected areas are represented by blue lines: 1. YardiRabe Supse Wildlife Sanctuary; 2. Mouling National Park; 3. Dibang Wildlife Sanctuary; 4. Mehao Wildlife Sanctuary; 5. Kamlang Wildlife Sanctuary; 6. Namdapha National Park; 7. Hponkanrazi Wildlife Sanctuary; 8. Hkakaborazi National Park; 9. Three Parallel Rivers of Yunnan Protected Areas.

The IUCN range of the Red Goral encompasses three countries: India, China, and Myanmar (Figure 1). In India, this species is recorded in specific regions of eastern Arunachal Pradesh, particularly in the districts of Upper Siang, Dibang Valley, Lower Dibang Valley, Anjaw, Lohit, and Changlang, including the Namdapha National Park (NNP) and the community forests near Vijaynagar, which borders with Myanmar. Additionally, there have been unconfirmed sightings near the River Kameng in western Arunachal Pradesh. In Myanmar, the Red Goral is found in the northern mountains of Kachin State, with a confirmed presence in Putao District’s Hponkanrazi Wildlife Sanctuary (HpWLS) and Hkakaborazi National Park (HkNP). Furthermore, China has the largest distribution area for this species, with populations confirmed in the Tibet Autonomous Region (Xizang) and Yunnan. In Tibet, the primary distribution area is Nyingchi City prefecture, while in Yunnan, the species is reported in Nùjiāng Lisu Autonomous Prefecture, specifically in Gongshan Derung and Nu Autonomous County [16]. Therefore, to achieve a comprehensive understanding of the distribution of the Red Goral, the entire IUCN extent was designated as the study area (Figure 1). This was conducted to ensure the analysis encompassed the full geographical range of the species and provided an accurate picture of its habitat distribution across different countries and regions aimed to identify the suitable habitats and their variations due to climate change within the IUCN known extent. Additionally, examining the IUCN extent is warranted because it employs a systematic process that entails mapping and analyzing a species’ geographical distribution using occurrence records, adjusting for geographical barriers, and verifying with expert knowledge to accurately reflect the species’ natural range for effective conservation planning and risk assessment.

The study utilized 14 location points sourced from the GeoCAT website, which compiles data from GBIF and iNaturalist, accessed on 25 June 2024 [33]. Furthermore, to ensure the reliability of the dataset and accurately represent the ecological areas of interest, records of museum specimens or captive individuals were excluded. Additionally, five location points were gathered during field visits to the temperate mountainous regions of Arunachal Pradesh, including sightings at Mayudia Pass (*n* = 1), the 65 km area in Dibang Valley (*n* = 2), Walong (near the cliff around 4 km from the airbase) (*n* = 1), and near Kaho in Anjaw district (*n* = 1). Moreover, 12 additional location points were obtained through direct communications with tourist guides and local people and hunters in Dibang Valley (near Anini), NNP, and Anjaw (near Kibitoo area) districts. The spatial correlation between occurrences was conducted at a resolution of 1 km^2^ in SDM toolbox v2.4. This specific resolution was chosen to align with the size of one pixel in the raster data, thereby reducing the risk of overfitting the model and ensuring a more accurate analysis. Consequently, the final model was generated using a total of 31 location points for the Red Goral within its IUCN extent.

### 2.2. Selection of Predictors for the Ensemble Model

The study employed various predictor sets, including bioclimatic, topographic, and habitat variables, to model the habitat of the Red Goral. The bioclimatic variables (*n* = 19) were sourced from the WorldClim website (https://www.worldclim.org/, accessed on 25 June 2024), while topographic variables, such as elevation, slope, and aspect, were obtained from the Diva-GIS website (http://srtm.csi.cgiar.org/srtmdata/, accessed on 25 June 2024) at a spatial resolution of 90 m [34]. The habitat variable, i.e., temperate forest (identified as of major importance by the IUCN), was extracted from the land cover classes in the Copernicus dataset and converted into continuous raster datasets using the Euclidean Distance function in ArcGIS [35]. This process aimed to evaluate the significance of this forest type and the species’ response to its proximity, as many species show a strong preference for areas near suitable forest types [36]. All variables were then resampled to 30 arcseconds (~1 km^2^) using the Spatial Analyst Extension in ArcGIS 10.6. The spatial multicollinearity testing was conducted using the SAHM (Software for Assisted Habitat Modelling) package in VisTrails software V.2, with covariates showing a correlation (r) greater than 0.8 being excluded from the analysis [37,38]. Furthermore, after addressing the correlation among the variables, a total of 8 variables were retained for the final model for both species (Figure S1).

### 2.3. SDM Utilizing Ensemble Approach

The assessment of distribution models involved employing multiple modeling algorithms through an ensemble approach to formulate the final distribution model for both species. Therefore, four distinct algorithms—Maximum Entropy (MaxEnt), Boosted Regression Tree (BRT), Generalized Linear Model (GLM), and Multivariate Adaptive Regression Splines (MARS)—were utilized [27,39,40] (Table S1). These algorithms were executed using the SAHM package in VisTrails software [37,41]. The execution produced probability surfaces ranging from “0” (least suitability) to “1” (highest suitability), and binary maps were generated using the minimum training presence as the threshold. The ensemble count map was constructed on a scale from “0” to “4”, where each pixel denoted the number of model agreements, with a value of “4” indicating unanimous agreement across all four models, facilitating habitat configuration analysis. Additionally, to assess and compare model performance, various evaluation metrics, including AUC, True Skill Statistic (TSS), Cohen’s Kappa, Proportion Correctly Classified (PCC), specificity, and sensitivity, were calculated for both the training data and cross-validation sets (*n* = 10) [42,43,44,45].

Furthermore, future projections were used to project potential climate change scenarios across two distinct shared socio-economic pathways (SSP)—namely SSP245 and SSP585—spanning the periods 2041–2060 and 2061–2080. The SSPs are scenarios used in climate change research to explore future socioeconomic conditions and their implications for greenhouse gas emissions and climate impacts. SSP245 represents a future where moderate efforts are made to mitigate emissions and adapt to climate change, assuming moderate population growth, technological development, and a balanced approach to environmental and social policies. Here, greenhouse gas emissions increase gradually over the 21st century, stabilizing towards the end of the century with international cooperation on climate policies, albeit with challenges in implementation [46]. Conversely, SSP585 depicts a future with high greenhouse gas emissions and limited adaptation efforts, assuming rapid population growth, high energy demand, and minimal environmental regulation, leading to continued emission increases throughout the century. This scenario reflects a world with little international cooperation on climate policies and insufficient societal efforts to mitigate emissions [47]. The study utilized the Hadley Centre Global Environment Model in Global Coupled Configuration 3.1 (HadGEM3-GC31 LL), the sixth Coupled Model Intercomparison Project (CMIP6) [48]. The selection of this General Circulation Model (GCM) was based on its recognized performance in South and Southeast Asia and its ability to capture temporal fluctuations and excel in representing temperature distribution, as evidenced by previous research [49,50]. For the present study, non-climatic raster data, including the habitat variable and topographic variables, remained constant due to the difficulty of projecting their change pattern in the future [51,52]. This deliberate decision aimed at isolating the impact of climate change on the objective of the study [53]. Additionally, to facilitate the development of an effective conservation action plan, a comprehensive assessment of habitat suitability was conducted on the transboundary protected areas (PAs), given the distinct legal framework for each country. This qualitative assessment of suitable habitats across different transboundary PAs in their distribution range was conducted using the zonal statistics function in ArcGIS v.10.6 [36,53,54,55].

### 2.4. Assessment of Habitat Shape Geometry and Connectivity

For the assessment of the qualitative and geometric characteristics of suitable patches in both current and projected future scenarios, various class-level metrics were employed using FRAGSTATS version 4.2.1 [56,57]. This specialized software for landscape ecology, urban planning, and environmental management analyzes spatial patterns in landscapes and ecosystems, providing a suite of metrics and indices to quantify and understand landscape structure and composition. The study encompassed metrics such as the number of patches (NP), aggregate index (AI), patch density (PD), largest patch index (LPI), edge density (ED), total edge (TE), and landscape shape index (LSI). Furthermore, metrics like NP, PD, ED, TE, and LPI provide detailed information about the geometry of the patches, including their size, edge characteristics, and density within a region. In contrast, LSI focuses on the shape complexity of the patches, indicating how convoluted or irregular they are, whereas AI measures the degree of proximity or clustering among patches, reflecting how aggregated or dispersed they are within the landscape. These metrics hold biological significance, shedding light on habitat ecological processes and offering valuable insights into the impacts of changes in suitable areas on landscape dynamics [58,59]. This methodology facilitates a deeper understanding of landscape characteristics and enables comprehensive analysis across the distribution range of the species. Consequently, these metrics were utilized to evaluate habitat features and levels of fragmentation in the modeled area across various scenarios, including present conditions and future climate change projections [60,61].

Furthermore, given the significance of enhancing habitat connectivity as a crucial conservation strategy for species preservation and gene flow, assessing the biological connectivity between habitat patches was imperative [36]. Therefore, to achieve this objective, the circuit model, commonly employed in designing animal corridors, was utilized [62]. The circuitscape toolbox for ArcGIS 10.6 facilitated the simulation of these corridors, where location points were used as nodes, with the conductance surface derived from the probability output generated by the ensemble model [60,63]. This toolbox facilitates the simulation of ecological corridors by modeling species movement or flow across diverse landscapes. In this process, pairwise source/ground mode settings were employed, where the probability maps generated from the model were utilized as the conductance raster. The location points were designated as the focal node raster in the pairwise setup module. The resulting output was generated as current maps, which were then used for further detailed assessment and analysis of connectivity. This corridor simulation was conducted for the present and all the future climatic scenarios.

## 3. Results

### 3.1. Ensemble Habitat Modeling

**Figure 2:**
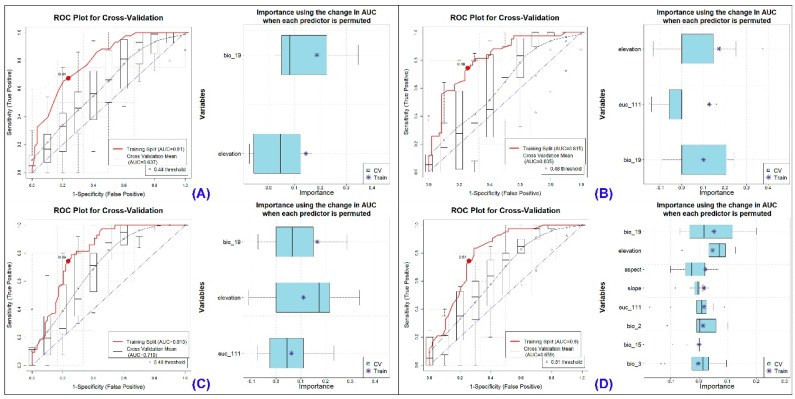
Model evaluation plot showing the average training ROC of both training and cross-validation (CV) and the predictors chosen by the model for the replicate runs under four models of Red Goral: (**A**) showing ROC plot of Boosted Regression Tree (BRT), (**B**) Generalized Linear Model (GLM), (**C**) Multivariate Adaptive Regression Spines (MARS), and (**D**) Maximum Entropy (MaxEnt).

The models exhibited an Area Under the Curve (AUC) range of 0.659 to 0.815 during training and between 0.637 and 0.719 in cross-validation for the Red Goral (Figure 2, Figures S2 and S3, Table 1). Notably, the ΔAUC value, which measures the difference between training and cross-validation AUCs, was the smallest for the MARS model, recording a value of 0.094. In contrast, the GLM demonstrated the highest ΔAUC values, reaching 0.18 across the replicate runs. These findings collectively underscore the sensitivity of the data employed for model fitting across all models. The evaluation metrics, including True Skill Statistic (TSS), Proportion Correctly Classified (PCC), Cohen’s Kappa, sensitivity, and specificity, further affirmed the high-quality performance of the models in both the training and cross-validation phases. Among the four selected models, MaxEnt utilized all provided variables during the replicate runs, whereas the BRT model opted for the fewest variables, selecting only two out of the eight provided for this species (Figure 2, Table 1).

### 3.2. Effective Habitat Suitability Predictors

The ensemble model for the Red Goral revealed that on average (μ) across the four models, the primary contributor to habitat suitability was the bioclimatic variable precipitation of the coldest quarter (bio_19), which accounted for 35.87% of the prediction power (Table 2, Figure S4). Furthermore, the topographic variable elevation was identified as the second most significant predictor, contributing 33.69%, whereas the habitat variable temperate forests (euc_111) emerged as the third highest predictor, accounting for 14.63% of the model ensemble run. In contrast, among the eight selected variables, the bioclimatic variable isothermality (bio_3) was the least influential in predicting the distribution of the Red Goral. 

### 3.3. Suitable Habitat Extent in the IUCN Extent and Protected Areas

**Figure 3:**
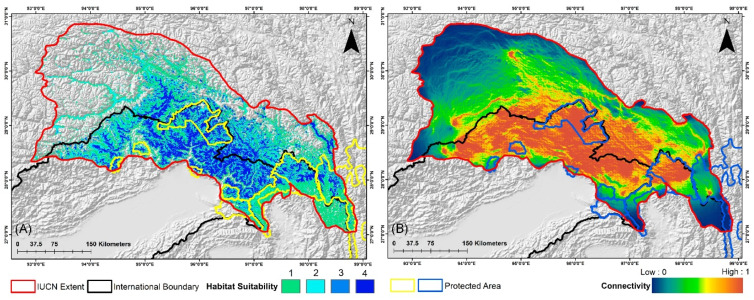
(**A**) This figure shows the present suitable habitats for *N. baileyi* in the study area. The four classes (1–4) defined in the map show the four model arguments used in the present study. Class “0” of habitat suitability is not indicated in the map as it represents no suitability and zero model agreement. (**B**) Map representing the habitat connectivity in the IUCN extent in the present scenario.

**Figure 4:**
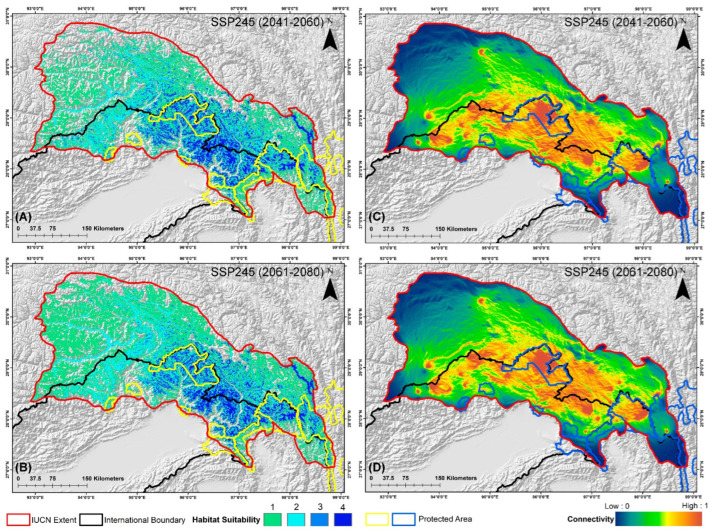
Maps representing the two-time frames of the SSP245 scenario for *N. baileyi*: (**A**,**B**) determine the habitat suitable in the IUCN extent, whereas (**C**,**D**) determine the connectivity in the landscape in these scenarios.

**Figure 5:**
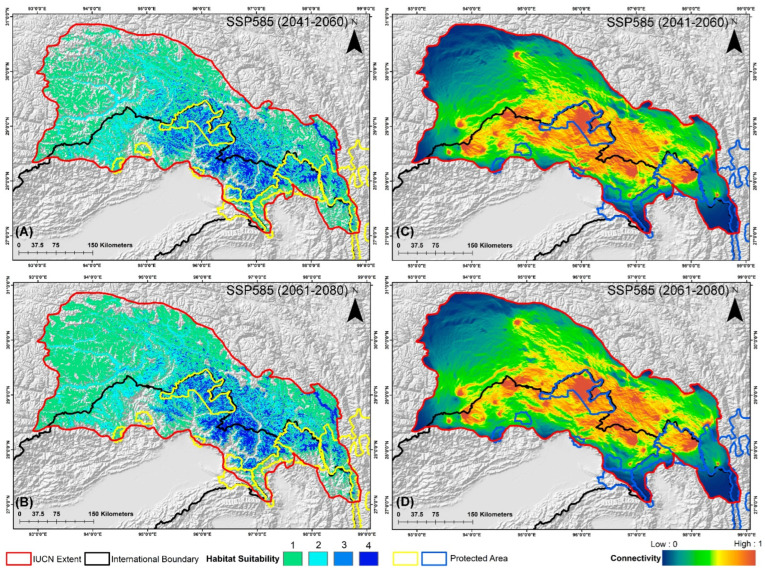
Maps representing the two-time frames of the SSP585 scenario for *N. baileyi*: (**A**,**B**) determine the habitat suitable in the IUCN extent, whereas (**C**,**D**) determine the connectivity in the landscape in these scenarios.

The estimated extent of occurrence for this species is reported to be 164,150 km^2^ within its distribution range, according to the IUCN. However, the findings of the ensemble model are concerning, revealing that only 21,363 km^2^, or a mere 13.01% of this area, represents suitable habitat under current conditions (Figure 3). The projections for future climate change scenarios present even more alarming trends, indicating a substantial reduction in suitable habitat that could reach up to 45% due to climatic shifts. Specifically, under the SSP245 scenario, the model predicts habitat declines of 34.82% for the period 2041–2060 and 41.55% for the period 2061–2080 (Figure 4, Table S2). Furthermore, the high emission scenario SSP585 forecasts even more severe reductions, with suitable habitat expected to decrease by 39.68% during 2041–2060 and by 45.74% during 2061–2080, compared to the present scenario, respectively (Figure 5, Table S2).

Additionally, nine transboundary PAs fall within the IUCN distribution range of this species across India, China, and Myanmar (Table 3). Despite its greater extent, only 4762 km^2^ of suitable habitat is included within the PAs, accounting for just 22.29% of the habitat identified by the model. Among them, Dibang Wildlife Sanctuary (DWLS) in India has the largest extent of suitable habitat at 1852 km^2^, while Mehao Wildlife Sanctuary (MWLS) in India has the smallest extent (71 km^2^) (Table 3). However, the assessment of mean habitat suitability yields different results as Yardi-Rabe Supse Wildlife Sanctuary (YRSWLS) in India has the highest mean habitat suitability score of 2.497, whereas Mouling National Park (MNP) has the lowest at 1.445 under current conditions (Table 4). Furthermore, the future scenarios predict a rapid decrease in both area extent and mean habitat suitability. Specifically, YRSWLS and MNP are projected to experience the most significant declines, exceeding 89% of area extent in both future scenarios for the 2041–2060 and 2061–2080 timeframes. Similarly, DWLS is projected to see a 35% decline in area extent under the SSP245 (2041–2060) scenario. In contrast, HkNP in Myanmar is expected to experience the least decline among all PAs, ranging from 9% to 15%. Interestingly, HpWLS is the only PA that shows an increase in area extent by over 4%, reaching 59.53% under the SSP245 (2061–2080) scenario. Furthermore, the SSP585 (2061–2080) scenario predicts the most severe declines compared to the other scenarios. Moreover, in terms of mean habitat suitability, MNP is expected to suffer the greatest decline, with an 87% decrease under the SSP585 (2061–2080) scenario. In contrast, DWLS is the only PA projected to see an increase in mean habitat suitability, up to 8%, due to climatic shifts. These projections underscore the pronounced declines and shifts caused by climate change, although the impacts are relatively less severe in the SSP245 (2041–2060) and SSP585 (2041–2060) scenarios compared to the 2061–2080 timeframe, which marks the onset of more significant climate-induced changes (Table 3 and Table 4).

### 3.4. Habitat Fragmentation and Biological Corridors

The future habitat loss induced by climate change has caused significant fragmentation in the suitable habitat patches for this species. The results reveal severe fragmentation of viable patches, as indicated by the NP metric, which increases by 26.39% in SSP245 (2041–2060) and 21.38% in SSP585 (2041–2060) compared to the present (Table 5). However, this rate slows to 17.75% and 13.55% in SSP245 (2061–2080) and SSP585 (2061–2080), likely due to greater overall habitat loss. Furthermore, the heightened fragmentation is reflected in increases of PD and ED by over 1658.93% and 23.14%, respectively, across all future scenarios, indicating increased density of the smaller patches with greater edge areas. Moreover, the LPI declines by over 38.02%, showing a substantial reduction in the size of viable patches, supported by the TE metric, which also declines between 17.91% and 25.53%, revealing the shrinkage of patches. Additionally, the decrease in both patch size and the number of patches explains the decline in the LSI by up to 6.77%, indicating simpler patch geometry due to reduced sizes as supported by the AI value, which decreases by more than 16%, revealing that patches are now farther apart. Overall, climate-induced shifts result in the fragmentation of currently viable patches into numerous smaller, more isolated patches, highlighting extensive habitat fragmentation (Table 5).

The assessment of biological corridors revealed the highest connectivity between DWLS and MWLS, with a mean connectivity value of 0.0583, followed by MNP and DWLS, with a mean connectivity value of 0.0538 in the present scenario (Figure 2, Table 6). Conversely, the lowest connectivity was found between Kamlang Wildlife Sanctuary (KWLS) and NNP, with a mean connectivity value of 0.0172, despite their close proximity to the landscape. Furthermore, the climate change projections indicate a decline in connectivity among the PAs in all future scenarios. Specifically, in the SSP245 scenario, the most significant decline of 25.60% from the present scenario was observed between YRSWLS and MNP during the 2041–2060 period. This decline was further compounded in the SSP245 (2061–2080) scenario, exceeding 31% (Figure 4). A similar trend of decline between YRSWLS and MNP was also observed in the SSP585 scenarios (Figure 5). However, three connectivity corridors between the PAs in this landscape showed relatively less decline in future projections. These include the corridor between HkNP and Three Parallel Rivers of Yunnan Protected Areas (TPRYPA) (decline by 1.2%), HpWLS and HkNP (decline up to 10%), and NNP and HpWLS (decline between 3.97% and 12.67%) (Table 6). Overall, the highest decline in connectivity was observed in the SSP585 (2061–2080) scenario, indicating that future climate change poses a significant threat to connectivity and, consequently, the conservation of the Red Goral. 

## 4. Discussion

Recent research has highlighted troubling trends in the global decline of mammalian species and populations, underscoring the severity of the situation [64]. This widespread decrease in mammalian diversity can largely be attributed to changes in land cover and environmental crises driven by climate change, particularly in South Asia [65]. Although numerous studies have been conducted worldwide to address these issues in various mammalian species, this work stands out as the first comprehensive analysis of the distribution, habitat suitability, and connectivity of viable patches for the Red Goral. The intricate details provided by this study not only fill a critical gap in understanding this particular species but also offer valuable insights that could guide future conservation efforts.

The findings reveal deeply concerning trends for the Red Goral, as the model identified only 21,363 km^2^, or a mere 13.01% of the total area, as suitable habitat within the IUCN extent under current conditions (Figure 3, Table S2). This limited, suitable habitat is alarming, as it leaves the species with very little space to thrive. Furthermore, this situation is compounded by the fact that only around 22.29% of this identified suitable habitat falls within the PAs, further constraining the species’ ability to survive in a protected landscape. The future projections paint an even grimmer picture, with a predicted decline of over 34% in suitable habitat extent. Given the restricted range of the species, this additional reduction due to climate change significantly worsens the situation. These findings are consistent with previous studies that concluded red gorals might face more severe challenges in the Tibetan Plateau adjoining the eastern Himalayas from the pronounced impacts of climate change [66,67]. Furthermore, the gorals are particularly vulnerable to habitat loss and environmental changes because of their limited movement range in restricted extent [68]. The alarming results underscore the necessity of enhancing research efforts focused on habitat modeling and identifying key predictors for species conservation. This type of research is especially critical for lesser-known ungulate species with very restricted ranges, such as the Red Goral [23]. Additionally, these research initiatives can provide valuable insights and inform conservation strategies to mitigate the adverse effects of habitat loss and climate change, ultimately aiding in the preservation of these vulnerable species.

Therefore, the present study identifies precipitation seasonality (bio_15) as the primary contributing predictor to the distribution of the Red Goral, accounting for 35.87% of the overall model (Table 2). This bioclimatic variable is closely followed by elevation, which is the second highest contributor, aligning with the observation that the species is predominantly found in the high-altitude mountains of the eastern Himalayas [16]. This finding corroborates previous studies conducted on various ungulate species, which have similarly highlighted the critical roles of precipitation and temperature as the top bioclimatic variables influencing their distribution [1,32]. Specifically, it was established that precipitation was the most influential variable in the distribution of ungulate species. This aligns with the results of the present study, where precipitation seasonality (bio_15) and precipitation of the coldest quarter (bio_19) are identified as significantly influential variables. However, the model in this study selected a different set of precipitation and temperature predictors compared to previous research, which could be attributed to the unique geographical context of the eastern Himalayas rather than the western ranges. Furthermore, elevation emerged as another key factor in predicting the habitat of the Red Goral, accounting for 33.69% of the overall model, suggesting that these animals may prefer higher-elevated temperate forests [10,69,70]. Therefore, the temperate forest variable (euc_111) also emerged as one of the most significant predictors for this species, contributing 14.63% to the prediction of the model.

The implications of these findings are particularly critical in the context of climate change in the future, as the distribution of the Red Goral is projected to shift towards the northeastern direction, corroborating the previous studies [20,71]. This northward shift in the Eastern Himalayan and Tibetan regions is clearly exhibited in the present study, emphasizing the need for proactive conservation efforts. Moreover, as the climate continues to change, it will be essential to focus conservation strategies on PAs that are likely to experience these shifts. Furthermore, ensuring that these PAs are well-prepared to accommodate the changing distribution of the Red Goral will be crucial for the species’ long-term survival. The study identifies nine designated transboundary PAs within the IUCN range of the Red Goral, highlighting their crucial role in supporting the species’ survival over time (Figure 3, Figure 4 and Figure 5). Among these, the DWLS in Arunachal Pradesh, India, is revealed as the PA with the largest extent of suitable habitat in the present scenario (Table 3). Additionally, HkNP in Myanmar also possesses a significant area of suitable habitat. These two PAs maintain considerable suitable habitat patches in future projections despite suffering some losses due to climatic shifts. This trend can be attributed to the anticipated northeastern shift in the distribution of Red Goral caused by climate change aligning with the geographic locations of these PAs and mitigating their losses to some extent. Moreover, the TPRYPA in China is projected to experience significant habitat loss. It is important to note that this PA is extensive, and only a small portion of it falls within the IUCN range of the Red Goral, which may explain discrepancies between the suitable habitat extent and mean habitat suitability results. The mean habitat suitability averages the quality of various habitats within the studied area, reflecting the overall potential for the species to thrive based on different environmental factors [72]. As a result, YRSWLS in India has the highest mean habitat suitability score (2.497) despite having a much smaller suitable extent (134 km^2^) than the other areas, which may be attributed to its relatively smaller size. This difference highlights the importance of considering both suitable extent areas and mean habitat suitability to gain a comprehensive understanding of the landscapes. It is notable that DWLS is unique in ranking high in both suitable extent and mean habitat suitability, making it a priority conservation area for the Red Goral. However, the entire distribution within the PAs is projected to be affected by climatic shifts, leading to a decline in both suitable habitat extent and habitat suitability scores. This trend results in significant fragmentation, especially in PAs like YRSWLS and MNP, where future scenarios predict a dramatic decline of over 90% in suitable areas. Additionally, the threat of irreversible biodiversity decline is particularly severe in these landscapes, which are already fragmented due to ongoing habitat loss from development activities. These activities reduce patch sizes and increase distances between patches, disrupting connectivity and exacerbating edge effects. The increased habitat fragmentation is linked to higher rates of species decline due to additional habitat loss [73]. The present study corroborates these findings, demonstrating that extensive reductions in suitable habitat have led to significant spatial alterations and fragmentation, adversely affecting the Red Goral and other ungulate species.

The distribution extent of the Red Goral has undergone extensive fragmentation due to climatic shifts (Table 5). This severe fragmentation of viable patches is evidenced by a notable increase in the NP metric by 26.39% in SSP245 (2041–2060) and 21.38% in SSP585 (2041–2060) when compared to the present scenario. These increases reflect a significant disruption in the continuity of the species’ habitat, indicating that climate change is causing the habitat to break apart into smaller, more isolated patches. However, this rate of increase in fragmentation reduces to 17.75% in SSP245 (2061–2080) and 13.55% in SSP585 (2061–2080). This deceleration in the rate of fragmentation may be attributed to the complete loss of some viable patches, which reduces the total number of fragments, indicating a critical threshold of habitat destruction. The increase in NP is also linked to the disintegration of suitable habitats, which has led to higher PD and ED in future scenarios while decreasing the LPI. This indicates that the fragmented patches have decreased in size and become more restricted for the species. Furthermore, the decrease in AI exacerbates the situation, as it signifies that these small, disintegrated patches will be far from each other, thus hindering the movement of the species. This fragmentation is particularly concerning because it could restrict gene flow among different populations of the Red Goral, potentially increasing inbreeding rates as these isolated patches are less likely to support viable populations over the long term [19,74]. Therefore, the identification and protection of corridor connectivity among fragmented habitats and PAs are crucial for the conservation of this species, as ensuring such connectivity will help maintain genetic diversity and facilitate the natural movement of the species, mitigating some of the adverse effects of habitat fragmentation induced by climate change.

The study assessed the corridors among the PAs within the Red Goral’s IUCN extent, identifying eight significant connectivity pathways (Table 6). It revealed that the highest mean connectivity (0.0583) was found between DWLS and MWLS in the present scenario, followed closely by the corridor between MNP and DWLS, with a mean connectivity of 0.0538. Conversely, the lowest connectivity was observed between KWLS and NNP, with a mean suitability score of 0.0172. This is particularly intriguing given their close proximity, highlighting the necessity of landscape-level connectivity. Furthermore, important transboundary corridors, such as the one between HkNP and the TPRYPA, with a mean connectivity score of 0.0493, and the corridor between NNP and HpWLS, with a mean connectivity score of 0.0332, were identified, which necessitates transboundary cooperation and collaboration. However, future scenarios project a decline in crucial corridors due to climatic shifts in the landscape level for this species. Notably, a significant decrease of over 25% in mean connectivity is anticipated between YRSWLS and MNP, whereas the corridor between HkNP and TPRYPA is projected to experience the least decline in mean connectivity, up to 1%. This minimal decline witnessed between HkNP and TPRYPA is likely due to their close proximity, underscoring the importance of maintaining continuous transboundary landscapes. The findings underscore the paramount importance of implementing conservation strategies to maintain and augment connectivity between the PAs to ensure the long-term viability of the Red Goral and other species that are dependent on these temperate mountain habitats. 

Thus, the PAs identified by this study require enhanced protection through implementation by governmental agencies; specifically, DWLS and HkNP, having the highest suitable extent, need immediate attention for conservation efforts. Additionally, considering the current sociopolitical situation in Myanmar, wildlife conservation efforts are severely jeopardized [75]. As a result, the study strongly urges international communities to support conservation efforts in these regions by providing aid to indigenous people and non-governmental organizations. In the Indian context, the construction of new hydropower dams in Arunachal Pradesh, particularly in Dibang Valley, has led to the clearing of many hectares of forest land. The study suggests conducting impartial environmental impact assessments (EIAs) before initiating any construction and insists that construction agencies adhere to EIA regulations while supporting local conservation efforts. Furthermore, traditional practices like trophy hunting and bushmeat hunting, embedded in tribal cultures due to historically high forest cover and low human presence, must be addressed. The recent development in these areas calls for awareness programs to reduce hunting practices and encourage engagement in conservation work supported by the growing tourism industry. Moreover, the TPRYPA in China requires a landscape-level assessment to identify habitats for this species that are not included in the IUCN extent. Furthermore, it is crucial to monitor and prevent the trade of body parts of Red Goral and other ungulates for medicine in the international market through these significant wildlife trafficking routes. Moreover, it is necessary to encourage the establishment of more captive breeding facilities for both breeding and research, especially since there is currently only one such facility at the Shanghai Zoo in China [76]. In order to effectively safeguard transboundary corridors, it is essential to maintain their continuity. When disruptions occur, it is important to implement measures, such as constructing underpasses or overpasses, to ensure better habitat connectivity. Achieving this goal requires collaboration and cooperation among the three countries involved. Moreover, forming community reserves with support from both international and national agencies is necessary for the long-term preservation of these vital corridors. Furthermore, a reassessment by the IUCN is also needed, potentially considering the species under the “Endangered” category, as this reclassification would help enhance protection and attention towards the species, which occupies a very restricted range and is significantly affected by climate change. Therefore, implementing these strategic measures will substantially enhance the conservation efforts for the Red Goral and other ungulate species.

## 5. Conclusions

In conclusion, this study offers a crucial, comprehensive analysis of distribution, habitat suitability, and connectivity, addressing significant gaps in understanding the vulnerable Red Goral. The findings reveal a troubling scenario with a very limited area identified as a suitable habitat under current conditions, and projections indicate a further decline due to climate change. This situation is exacerbated by the limited protection within designated areas, with only a small portion of suitable habitats falling under PAs. The identification of key PAs, such as DWLS in India and HkNP in Myanmar, as priority regions for conservation efforts is particularly significant for this species. Additionally, the study emphasizes the importance of maintaining and enhancing connectivity among fragmented habitats and transboundary corridors to mitigate the adverse effects of habitat fragmentation and climate change. However, a more exhaustive field survey on the species outside of the known IUCN extent will help to expand the training area for further SDM research to understand the climate change shift of the species on a broader scale across this temperate mountainous region. The recommendations for immediate conservation actions, including international support, stricter adherence to EIA, and addressing traditional hunting practices, are essential steps towards safeguarding the Red Goral. The insights provided a vital foundation for future research and conservation initiatives, aiming to ensure the long-term survival of this ungulate species in its natural habitat.

## Figures and Tables

**Table 1:** Model fit metrics for each of the participating modeling methods and for the final ensemble model for estimation of habitat suitability of *N. baileyi*. A total of four model algorithms i.e., Maximum Entropy (MaxEnt), Boosted Regression Tree (BRT), Generalized Linear Model (GLM), and Multivariate Adaptive Regression Splines (MARS). AUC: Area under Curve, ΔAUC: Change in Area under Curve (Training–Cross Validation), PCC: Proportion Correctly Classified, TSS: True Skill Statistic.

Species	Model	Dataset	AUC	ΔAUC	PCC	TSS	Kappa	Specificity	Sensitivity
*Naemorhedus baileyi*	BRT	Train	0.81	0.173	72.5	0.437	0.437	0.763	0.674
		CV	0.637		64.7	0.275	0.273	0.68	0.595
	GLM	Train	0.815	0.18	74.5	0.49	0.484	0.746	0.744
		CV	0.635		59.2	0.157	0.165	0.657	0.5
	MARS	Train	0.813	0.094	75.5	0.507	0.502	0.763	0.744
		CV	0.719		65.3	0.288	0.284	0.683	0.605
	MaxEnt	Train	0.8	0.141	74.3	0.486	0.48	0.741	0.744
		CV	0.939		88.1	0.746	0.697	0.887	0.859

**Table 2:** The mean percentage contribution of each covariate generated from the final ensemble model for *N. baileyi*.

Variable	Abbreviation	BRT	GLM	MARS	MaxEnt	μ (Mean)	μ (Mean) %
Aspect	aspect	0.000	0.000	0.000	0.023	0.006	1.62
Precipitation Seasonality	bio_15	0.000	0.000	0.000	0.011	0.003	0.76
Precipitation of Coldest Quarter	bio_19	0.186	0.100	0.167	0.052	0.126	35.87
Mean Diurnal Range (Mean of Monthly (Max Temp Min Temp)	bio_2	0.000	0.000	0.000	0.014	0.003	0.98
Isothermality	bio_3	0.000	0.000	0.000	0.003	0.001	0.23
Elevation	elevation	0.144	0.172	0.111	0.047	0.119	33.69
Temperate Forest	euc_111	0.000	0.127	0.062	0.017	0.052	14.63
Slope	slope	0.000	0.000	0.000	0.172	0.043	12.21

**Table 3:** The suitable habitat extent (in km^2^) for *N. baileyi* in both current and future scenarios within protected areas (descending order) in the IUCN extent. The gain is represented by “+”, whereas loss is represented by “−”. WLS: wildlife sanctuary, NP: national park, PA: protected area, and GR: growth rate.

Country	Protected Areas	Present	SSP 245 (2041–2060)	GR from Present (%)	SSP 245 (2061–2080)	GR from Present (%)	SSP 585 (2041–2060)	GR from Present (%)	SSP 585 (2061–2080)	GR from Present (%)
India	Dibang WLS	1852	1443	−22.084	1195	−35.475	1252	−32.397	1304	−29.590
Myanmar	Hkakaborazi NP	1191	1081	−9.236	1050	−11.839	1129	−5.206	1004	−15.701
China	Three Parallel Rivers of Yunnan PA	825	607	−26.424	514	−37.697	700	−15.152	592	−28.242
Myanmar	Hponkanrazi WLS	257	269	+4.669	410	+59.533	331	+28.794	281	+9.339
India	Kamlang WLS	210	120	−42.857	116	−44.762	109	−48.095	79	−62.381
India	Yardi-Rabe Supse WLS	134	14	−89.552	2	−98.507	4	−97.015	2	−98.507
India	Namdapha NP	126	120	−4.762	115	−8.730	112	−11.111	93	−26.190
India	Mouling NP	96	1	−98.958	0	−100.000	0	−100.000	0	−100.000
India	Mehao WLS	71	31	−56.338	27	−61.972	27	−61.972	10	−85.915

**Table 4:** The mean habitat suitability for *N. baileyi* in both current and future scenarios within protected areas (descending order) in the IUCN extent. The gain is represented by “+”, whereas loss is represented by “−”. WLS: wildlife sanctuary, NP: national park, PA: protected area, and GR: growth rate.

Country	Protected Areas	Present	SSP 245 (2041–2060)	GR from Present (%)	SSP 245 (2061–2080)	GR from Present (%)	SSP 585 (2041–2060)	GR from Present (%)	SSP 585 (2061–2080)	GR from Present (%)
India	Yardi-Rabe Supse WLS	2.497	1.081	−56.705	0.976	−60.911	1.147	−54.075	0.755	−69.763
India	Dibang WLS	2.361	2.225	−5.759	2.212	−6.315	2.231	−5.498	2.161	−8.461
India	Kamlang WLS	2.059	1.400	−31.996	1.322	−35.800	1.315	−36.113	1.011	−50.912
India	Mehao WLS	2.028	1.284	−36.682	0.905	−55.374	1.000	−50.701	0.616	−69.626
Myanmar	Hkakaborazi NP	2.028	1.735	−14.425	1.886	−6.996	1.796	−11.430	1.628	−19.716
India	Namdapha NP	1.905	1.356	−28.781	1.253	−34.230	1.431	−24.858	0.956	−49.811
China	Three Parallel Rivers of Yunnan PA	1.784	1.484	−16.823	1.659	−7.032	1.556	−12.787	1.339	−24.975
Myanmar	Hponkanrazi WLS	1.710	1.201	−29.752	1.282	−25.051	1.226	−28.320	0.841	−50.836
India	Mouling NP	1.445	0.246	−82.977	0.244	−83.079	0.396	−72.579	0.180	−87.564

**Table 5:** Assessment of habitat shape geometry of *N. baileyi* in present and future scenarios. NP: no. of patches, PD: patch density, LPI: largest patch index, ED: edge density, TE: total edge, LSI: landscape shape index, AI: aggregation index.

Scenario	NP	PD	LPI	TE	ED	LSI	AI
Present	1239	96,210,504.14	16.135	236.352	1,728,689.791	50.416	65.878
SSP 245 (2041–2060)	1566	1,757,433,743	14.473	194.032	2,177,512.031	51.386	56.932
SSP 245 (2061–2080)	1459	1,825,941,129	11.782	177.648	2,223,267.922	49.567	56.037
SSP 585 (2041–2060)	1504	1,823,684,619	13.651	180.944	2,194,047.804	49.601	56.620
SSP 585 (2061–2080)	1407	1,692,277,346	10.854	176.992	2,128,781.464	48.518	57.933

**Table 6:** Assessment of corridor connectivity of *N. baileyi* among the protected areas in both the current and future climate change scenarios. The gain is represented by “+”, whereas loss is represented by “−”. YRSWLS: Yardi-Rabe Supse Wildlife Sanctuary, MNP: Mouling National Park, DWLS: Dibang Wildlife Sanctuary, MWLS: Mehao Wildlife Sanctuary, KWLS: Kameng Wildlife Sanctuary, NNP: Namdapha National Park, HpWLS: Hponkanrazi Wildlife Sanctuary, HkNP: Hkakaborazi National Park, TPRYPA: Three Parallel Rivers of Yunnan Protected Areas, GR: growth rate.

Corridors	Present	SSP 245 (2041–2060)	GR from Present (%)	SSP 245 (2061–2080)	GR from Present (%)	SSP 585 (2041–2060)	GR from Present (%)	SSP 585 (2061–2080)	GR from Present (%)
YRSWLS_MNP	0.0426	0.0317	−25.60	0.0292	−31.35	0.0310	−27.15	0.0255	−40.11
MNP_DWLS	0.0538	0.0481	−10.57	0.0460	−14.54	0.0477	−11.39	0.0430	−20.02
DWLS_MWLS	0.0583	0.0532	−8.81	0.0505	−13.40	0.0516	−11.51	0.0468	−19.86
DWLS_KWLS	0.0446	0.0401	−10.12	0.0383	−14.10	0.0390	−12.60	0.0348	−22.09
KWLS_NNP	0.0172	0.0151	−12.05	0.0147	−14.74	0.0147	−14.29	0.0128	−25.84
NNP_HpWLS	0.0332	0.0316	−4.88	0.0319	−3.97	0.0316	−4.84	0.0290	−12.67
HpWLS_HkNP	0.0482	0.0461	−4.45	0.0466	−3.32	0.0461	−4.35	0.0434	−10.05
HkNP_TPRYPA	0.0493	0.0490	−0.49	0.0487	−1.22	0.0490	−0.55	0.0494	0.29

## Data Availability

Data used for the analysis were sourced from open-access resources.

## Supplementary Materials

The following supporting information can be downloaded at: https://www.mdpi.com/article/10.3390/biology13090667/s1, Figure S1. Figure showing the correlation between the covariates chosen for the final model for *N. baileyi*; Figure S2. Evaluation matrix performance across model runs for *N. baileyi*. Brown—represents the correlation coefficient among the four different models; Yellow—represents the proportion of deviance explained; Green—represents the proportion of correctly classified; Blue—represents Area under Curve (AUC); and Pink—represents True Skill Statistic. Figure S3. Confusion matrixes, model calibration plots, and residual plots for *N. baileyi*. Row 1 represents spatial pattern of residuals, where the color ramp indicates the magnitude of deviance and size represents the quantity. Row 2 represents the model calibration plot across all four different models for cross-validation split. Row 3 represents the confusion matrix for all four models, plotted by observed vs. predicted, where the color ramp from the lowest value of 0% (white) to 100% (red) indicates the quantification of particular pair types. Column a. represents plots for BRT, Column b. represents plots for GLM, Column c. represents plots for MARS, and Column d. represents plots for MaxEnt. Figure S4. The response curves of the covariates selected by each of the participating ensemble models for *N. baileyi*. (A) BRT, (B) GLM, (C) MARS, and (D) MaxEnt. Table S1. The details of the models used in the SAHM (Software for Assisted Habitat Modelling) package in VisTrails software; Table S2. The total suitable habitat extent of *N. baileyi* in the present and future climate change scenarios within its IUCN extent.
