# Supplementary material for: Habitat Loss in the IUCN Extent: Climate Change-Induced Threat on the Red Goral (Naemorhedus baileyi) in the Temperate Mountains of South Asia
Source: Biology (Basel). 2024 Aug 27;13(9):667. doi: 10.3390/biology13090667 (PMC11428959; doi:10.3390/biology13090667)
Supplement: Supplementary file 1 [file biology-13-00667-s001.zip › biology-3154674-supplementary.pdf]

Supplementary Materials

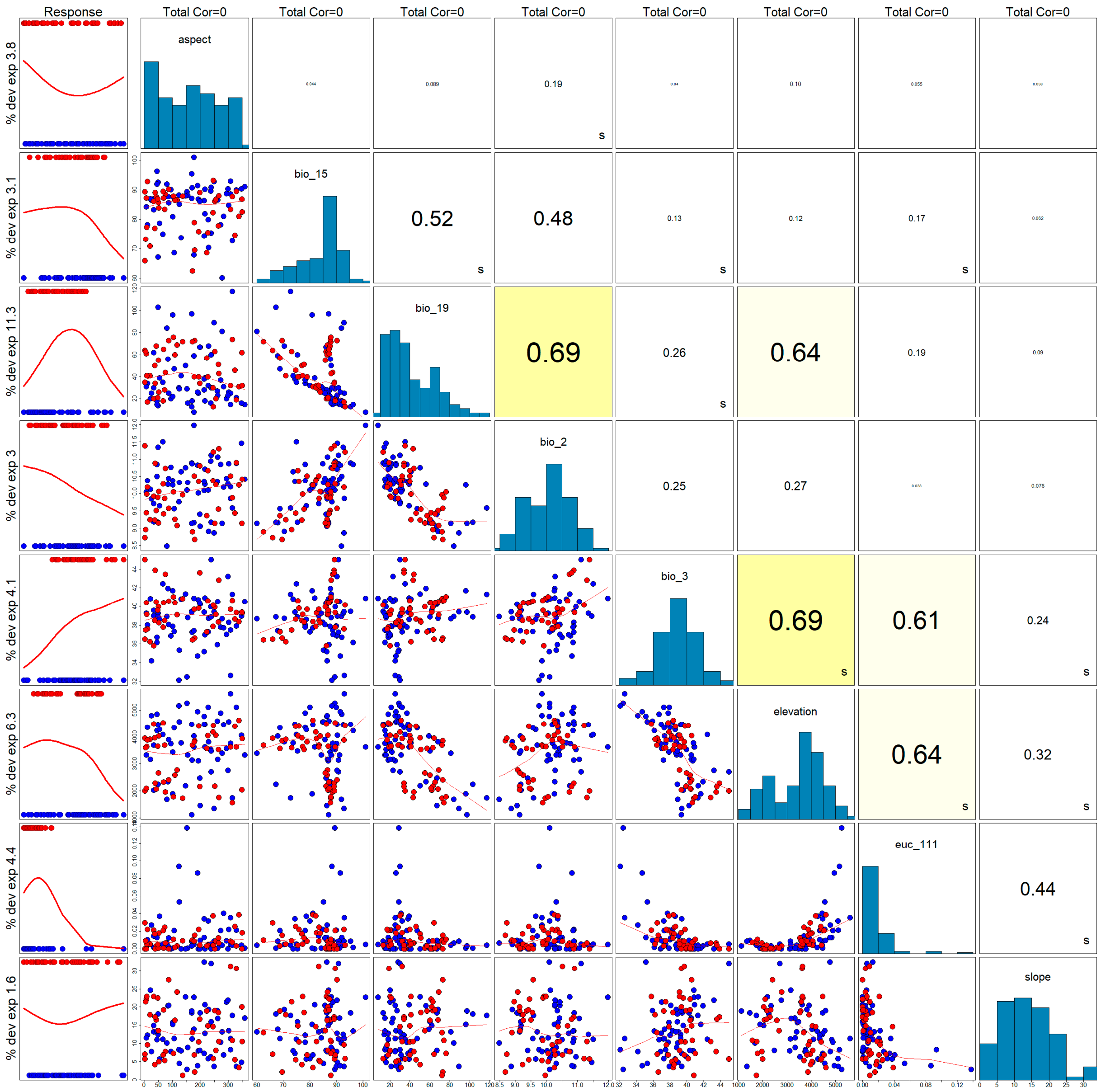

Figure S1. Figure showing the correlation between the covariates chosen for final model for *N. baileyi*.

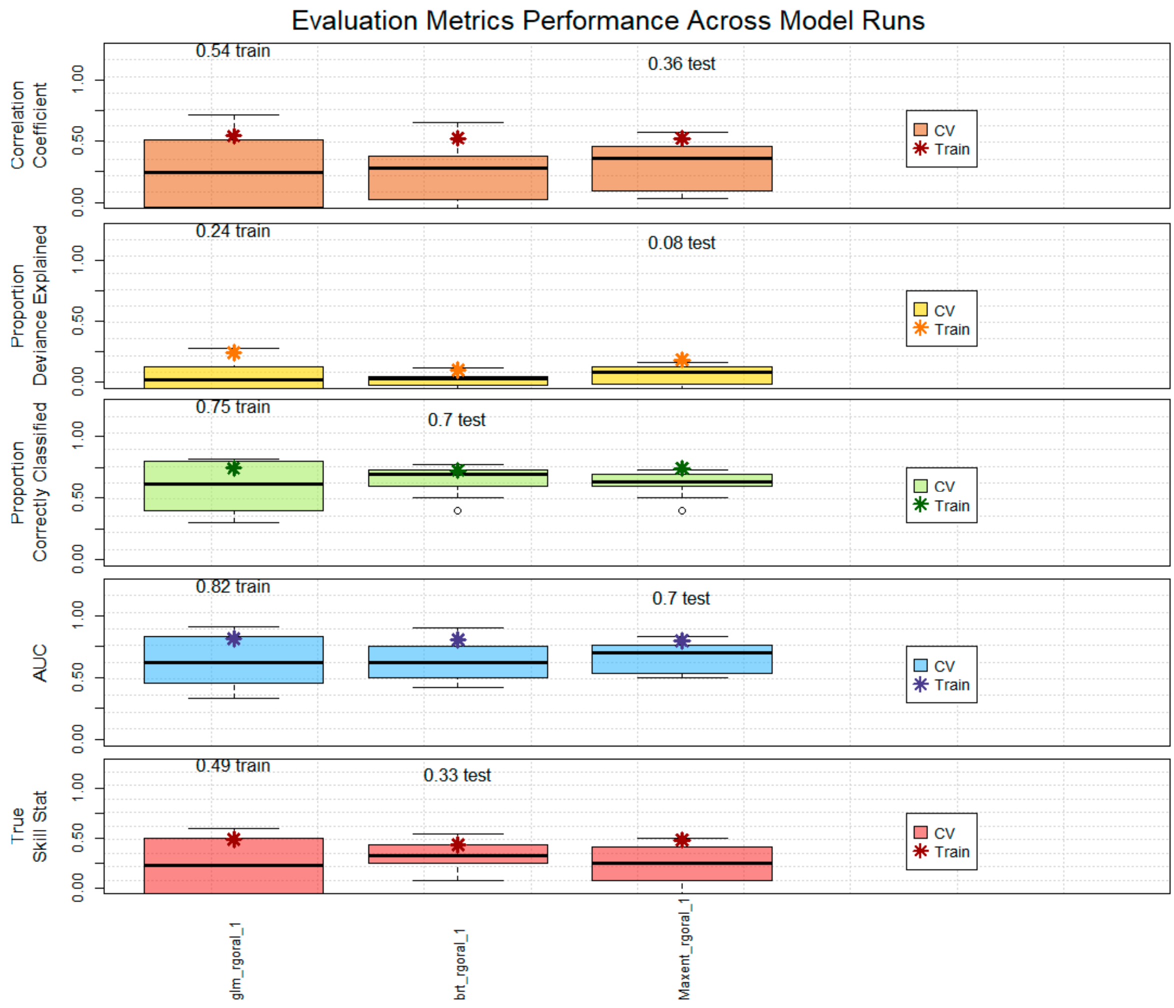

Figure S2. Evaluation Matrix performance across model runs for *N. baileyi*. Brown - represents the correlation coefficient among the four different models. Yellow - represents the proportion of deviance explained; Green - represents the Proportion of correctly classified; Blue - represents Area under curve (AUC) and Pink - represents true skill statistics.

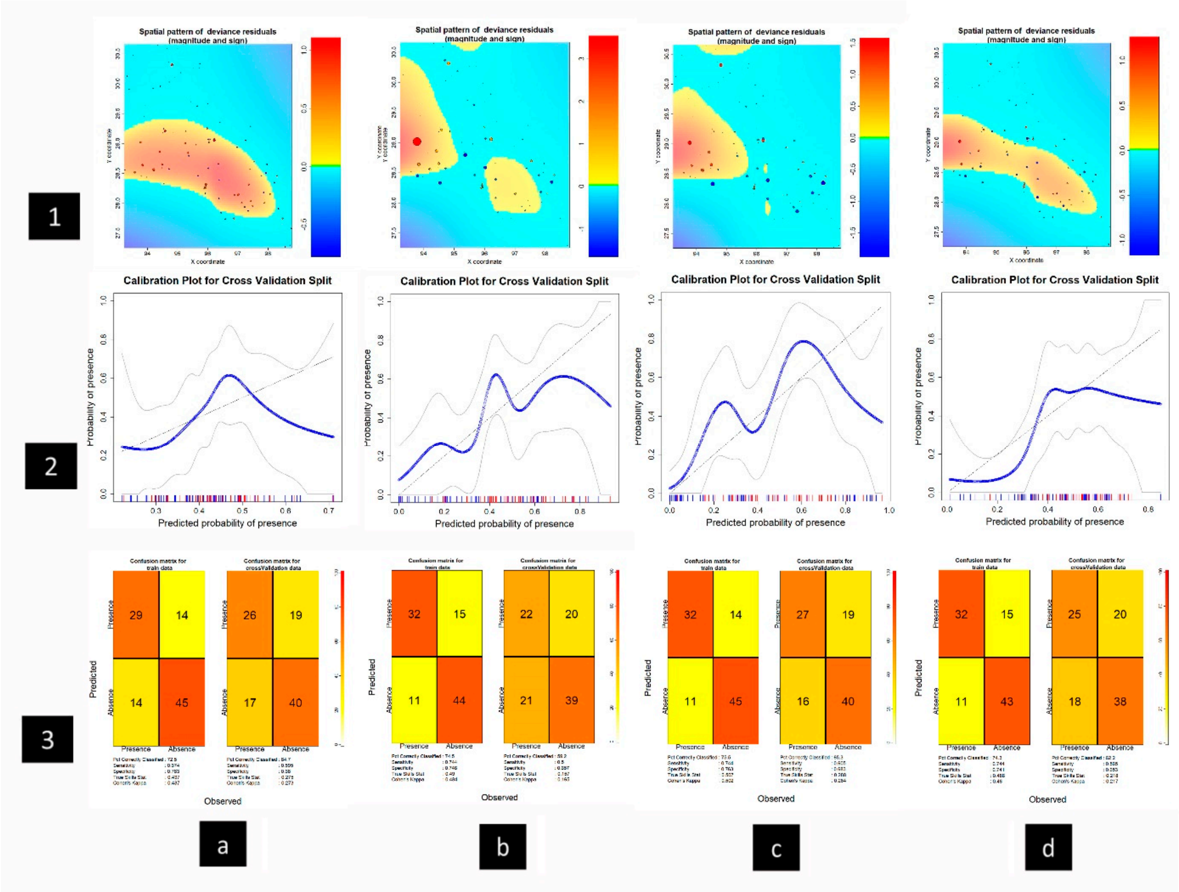

Figure S3. Confusion matrixes, Model Calibration plots and Residual plots for *N. baileyi*. Row 1 represents spatial pattern of residuals where colour ramp indicates the magnitude of deviance and size represents the quantity. Row 2 represents model calibration plot across all four different model for cross-validation split. Row 3 represents confusion matrix for all four models, plotted by observed vs. predicted where colour ramp from lowest value 0% (white) to 100% (red) indicates the quantification of particular pair types. Column a. represents plots for BRT, Column b. represents plots for GLM, Column c. represents plots for MARS, Column d. represents plots for MaxEnt.

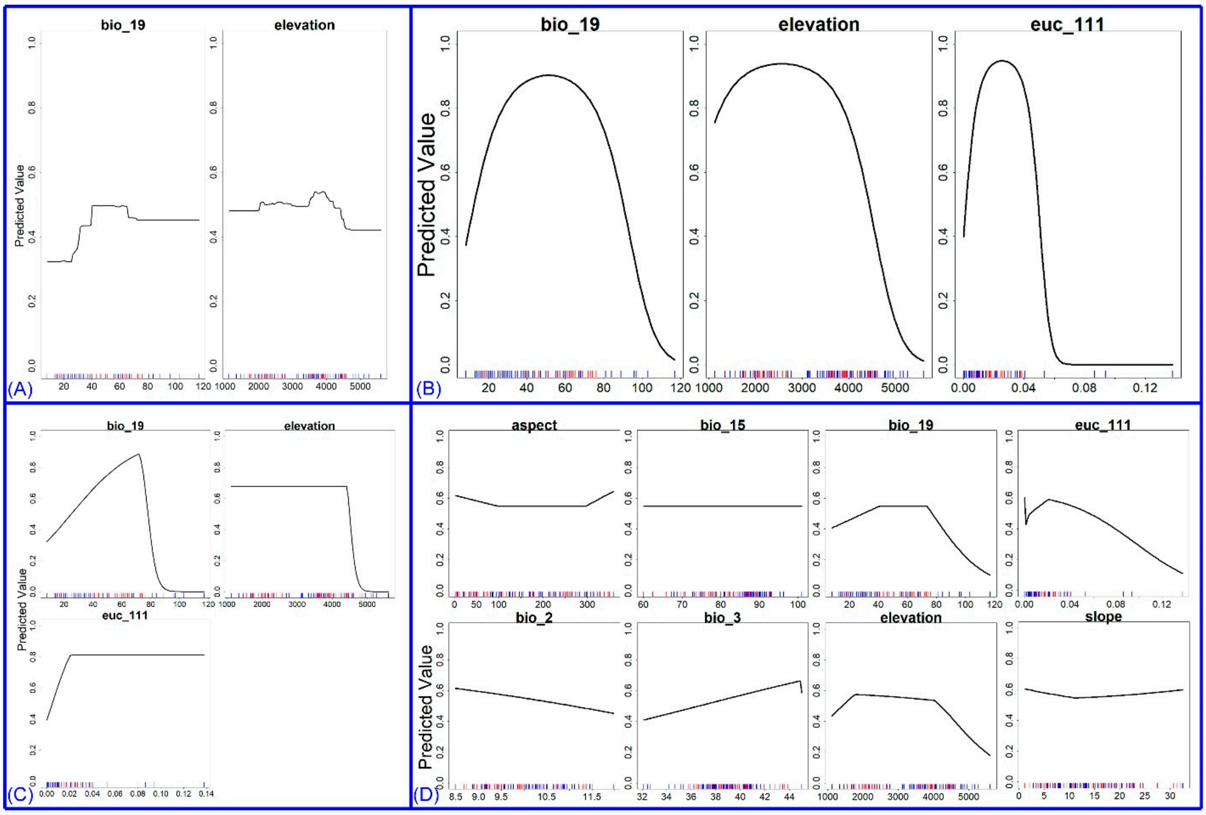

Figure S4. The response curves of the covariates selected by each of the participating ensemble model for *N. baileyi*. (A) BRT, (B) GLM, (C) MARS, (D) MaxEnt.

Table S1. The details of the models used in the SAHM (Software for Assisted Habitat Modelling) package in VisTrails software.

Models	Model Specific Settings
Boosted Regression Tree (BRT)	• makeMESMap: Boolean<br>• Alpha: 1.0 (Float)<br>• BagFraction: 0.75 (Float)<br>• NumberOfFolds: 3<br>• PrevalenceStratify: Boolean<br>• Seed: 1234<br>• SelectBestPredSubset: Boolean<br>• ThresholdOptimizationMethod: Sensitivity=Specificity<br>• Tolerance: 0.001<br>• ToleranceMethods: auto<br>• makeBinMap: Boolean<br>• makeProbabilityMap: Boolean
Generalized Linear Model (GLM)	• makeMESMap: Boolean<br>• SelectBestPredSubset: Boolean<br>• SimplificationMethod: AIC<br>• SquaredTerms: Boolean<br>• ThresholdOptimizationMethod: Sensitivity=Specificity<br>• makeBinMap: Boolean<br>• makeProbabilityMap: Boolean
Multivariate Adaptive Regression Splines (MARS)	• makeMESMap: Boolean<br>• MarsDegree: 1<br>• MarsPenalty: 2.0<br>• ThresholdOptimizationMethod: Sensitivity=Specificity<br>• makeBinMap: Boolean<br>• makeProbabilityMap: Boolean
Maximum Entropy (MaxEnt)	• ThresholdOptimizationMethod: Sensitivity=Specificity<br>• useRMetrics: Booelan<br>• autofeature: Boolean<br>• beta_threshold: -1.0<br>• jackknife: Boolean<br>• makeMESMap: Boolean<br>• makeBinMap: Boolean<br>• makeProbabilityMap: Boolean<br>• maximumbackground: 10000

	• maximumiterations: 5000
Random Forest (RF)	• makeMESMap: Boolean<br>• Seed: 1234<br>• ThresholdOptimizationMethod: Sensitivity=Specificity<br>• mTry: 1<br>• makeBinMap: Boolean<br>• makeProbabilityMap: Boolean

Table S2. The total suitable habitat extent of *N. baileyi* in present and future climate change scenario within its IUCN extent.

Scenario	Area (sq. km.)
Present	21363
SSP 245 (2041-2060)	13923
SSP 245 (2061-2080)	12485
SSP 585 (2041-2060)	12886
SSP 585 (2061-2080)	11591
